# Broncho-biliary fistula caused by a left hydatic cyst: a case report

**DOI:** 10.1016/j.ijscr.2025.111770

**Published:** 2025-08-07

**Authors:** Achref SARRAJ, Mohamed Ben KHALIFA, Firas JAOUED, Mossab GHANNOUCHI, Wassim El GUEDR, Moez BOUDOKHANE

**Affiliations:** Department of Surgery, Taher Sfar Hospital Mahdia, Monastir University School of Medicine, Tunisia

**Keywords:** Hydatid cyst, Left biliobronchial fistula, Bilioptysis, Cystobronchial fistula, Case report

## Abstract

**Introduction and importance:**

Broncho-biliary fistula (BBF) of hydatid origin is a rare complication due to an abnormal communication between the biliary tract and bronchial tree, affecting abdominal, diaphragmatic, and thoracic levels. Although BBF is already infrequent, its occurrence on the left side is exceedingly rare and scarcely documented in the literature.

**Case presentation:**

We report a 34-year-old woman with left-sided BBF caused by a segment II hepatic hydatid cyst (60 × 82 mm), presenting with bilioptysis and pneumonia resistant to antibiotics. Initial laboratory tests revealed marked hepatic cytolysis and cholestasis, with no clinical improvement despite appropriate antibiotic therapy with levofloxacin 500 mg twice daily for 14 days. CT revealed a type IB BBF per Mestiri's classification. Surgical management via laparotomy included cystectomy, fistula closure, and diaphragmatic repair. Operative time was approximately 135 min. There were no intraoperative complications.

**Discussion:**

This case illustrates a rare left-sided BBF managed effectively via an abdominal approach alone, avoiding thoracotomy. The decision was based on lesion accessibility, cyst location, and avoidance of gastric injury.

**Conclusion:**

This case emphasizes the importance of precise preoperative imaging and highlights that laparotomy alone may be sufficient in selected left-sided BBF cases, avoiding more invasive approaches like thoracotomy.

## Highlights

• Broncho-biliary fistula (BBF) of hydatid origin is a rare complication.
• Left-sided localization is even rarer.
• The treatment is primarily surgical.
• The choice of surgical approach must be adapted to the lesions at different levels, especially considering the type of BBF.

## 1 Introduction

Hydatid disease, an infection caused by the ingestion of eggs from the dog tapeworm Echinococcus granulosis, is commonly found in areas with poor sanitation and frequent human-animal interaction. The liver is the primary site of involvement (50–90 %), particularly affecting the right lobe. The lungs are also frequently involved, and in rare instances, the kidneys, brain, or other regions may be affected [1]**.**

As the hydatid cyst matures in the liver, it can give rise to various complications. One common complication is the rupture of hydatid cysts into the biliary tract, occurring at a frequency of 17 to 44 %. Among these complications, intrathoracic rupture is observed, occurring in 0.6 % to 16 % of cases. A broncho biliary fistula (BBF), though severe due to the multiplicity of lesions, is a less frequent complication primarily characterized by the presence of bile in the sputum, a condition known as bilioptysis [1,2]**.**

The primary mechanical factors contributing to the transdiaphragmatic migration of the cyst include positive abdominal and negative intrathoracic pressures, the vacuum-like action of diaphragmatic movements, and the traumatic effect of the cyst on surrounding structures.

The main cause of bronchial erosion and subsequent broncho biliary fistula (BBF) formation is the combination of pulmonary inflammation and the necrotizing action of bile [1]. According to Mestiri's classification, thoracic migrations of hepatic hydatid cysts are categorized into four types and nine subgroups: Type I (direct cyst-bronchus fistula), subdivided into IA (small-caliber) and IB (large-caliber); Type II (intrapulmonary cavities), Type III (intermediate pleural pouch), and Type IV (rupture into pleural cavity). Our case corresponds to type IB, characterized by a large-caliber bronchial fistula.

While surgery is universally accepted as the optimal treatment for BBF, debates continue regarding the most effective surgical approach and technique. Given the extreme rarity of such cases in the literature, this report offers significant scientific and academic merit and may provide surgeons with a valuable point of reference for comparable situations. We present a case and the work was reported in line with the SCARE criteria [3].

## 2 Case presentation

A 34-year-old female patient, with no notable pathological history, initially presented with abdominal pain, jaundice, and fever, which were ascribed to Hepatitis A. Upon reflection, it was determined to be a case of angiocholitis that resolved spontaneously. The patient is a Tunisian female of North African ethnicity, residing in a rural area where hydatid disease is endemic. She was a non-smoker, with a body mass index (BMI) within the normal range, and worked as a farmer. She later required a hospitalization in the pulmonology department for left Basi thoracic pain accompanied by fever, and a productive cough yielding bitter greenish sputum with a left basal pulmonary infiltrate on standard chest radiography. Initial laboratory workup revealed elevated bilirubin levels (total bilirubin: 57 μmol/L, conjugated bilirubin: 32 μmol/L), AST at 10 times and ALT at 7 times the upper normal limit. No sputum analysis was performed.

**Fig. 1:**
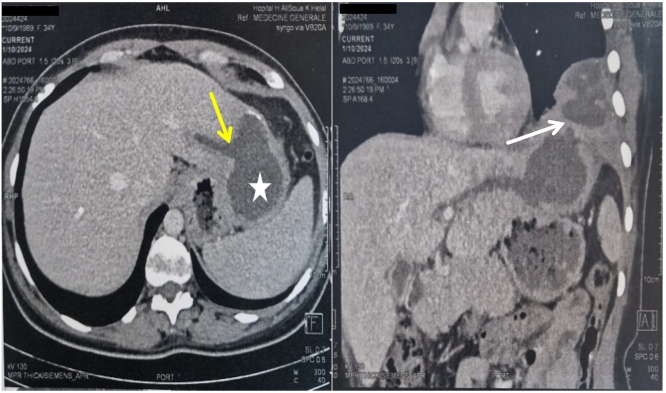
Thoraco-abdominal CT scan showing a 60 × 82 mm hydatid cyst in segment II of the liver (star) that communicated with the dilated left intrahepatic bile ducts via a 6 mm fistula (yellow arrow) and a large bronchial fistula(white arrow). (For interpretation of the references to colour in this figure legend, the reader is referred to the web version of this article.)
● Preoperative consultation with a thoracic surgeon was conducted. The surgeon concluded that thoracic involvement was minimal and thoracotomy was not required.
  Following adequate preparation, she underwent a left subcostal surgical approach. Surgical exploration identified a 70 mm hydatid cyst in segment II, causing displacement of the lesser gastric curvature and establishing a communication with the left hemithorax through a 40 mm diaphragmatic breach. Importantly, no additional phrenectomy was required, as the fistula was successfully managed through the existing diaphragmatic defect (Fig.2).

● Despite appropriate antibiotic therapy with levofloxacin 500 mg every 12 h for 14 days, the lack of clinical improvement and radiological clearance led to a thoracoabdominal CT scan. It demonstrated a 60 × 82 mm well-defined cystic lesion within the left hepatic lobe, characterized by a thickened wall and heterogeneous internal contents, findings highly suggestive of a complicated hydatid cyst. The cyst communicated with the dilated left intrahepatic bile ducts via a 6 mm fistula (Fig.1) and with the left lung through a diaphragmatic breach of 31 mm. Additional findings included localized pleural thickening adjacent to the lesion, without associated pleural effusion. This was classified as a type IB broncho biliary fistula according to Mesteri's classification [4].

**Fig. 2:**
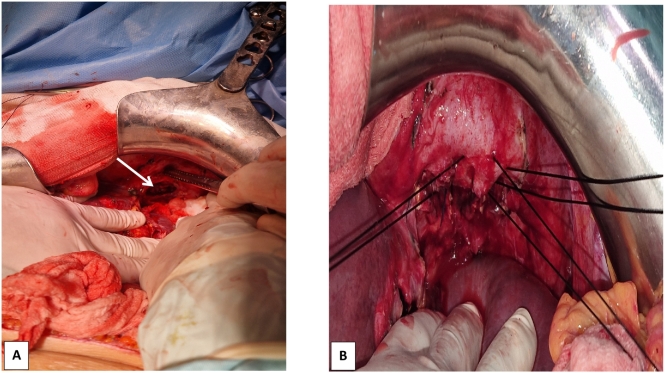
**A:** Intraoperative view showing a cystobronchial fistula within the diaphragmatic breach (arrow). **B:** intraoperative image revealing the suturing of the breach with non absorbable interrupted sutures.
  The procedure involved a cysto-diaphragmatic disconnection, followed by resection of the protruding dome after aspirating a biliary fluid from the cyst. This revealed a 6 mm cysto-biliary fistula that was sutured after confirming the absence of hydatid material in the non-dilated bile ducts via intraoperative cholangiography. Operative time was 135 min. The surgical team consisted of a senior hepatobiliary surgeon and two assistants. No intraoperative complications occurred. Closure of the bronchial fistula was achieved using absorbable 3–0 sutures. Diaphragmatic breach was repaired with non-absorbable interrupted sutures. Drains (Redon) were placed in the subhepatic and left pleural spaces. No cardiac complications occurred, and no intercostal drainage tube (ICDT) was necessary.
  Albendazole 400 mg twice daily was given for 4 weeks pre- and 3 months post-operatively.
  The postoperative course was uneventful, and a thoraco-abdominal CT scan at one month then at 6 month postoperatively showed regression of lesions with no hydatid recurrence. The patient remains under clinical and radiological follow-up beyond six months to monitor for late recurrence. The chronological sequence of clinical events is outlined in Table 1.

**Table 1:** Patient Timeline.

Week 0	➝	Week 1	➝	Week 3	➝	Week 4	➝	Week 8
Onset of symptoms (abdominal pain, jaundice)		Initial diagnosis of Hepatitis A		Admission to pulmonology for resistant pneumonia		**CT confirmed BBF;** **surgery performed**		Postoperative CT showed full regression

## 3 Discussion

Broncho biliary fistula (FBB) is an abnormal communication between the biliary tract and the bronchial tree. It represents a dreaded complication of hepatic hydatid cysts rupturing into the thorax due to the potential bronchopulmonary and hepatobiliary injuries it can cause. Hydatid cysts are localized in the right lobe in 67 % of cases, and typically, these broncho biliary fistulas arise from hydatid cysts located in the hepatic dome. However, in our patient, the broncho biliary fistula is due to a hydatid cyst in the left lobe of the liver, rendering it a unique and rare case [1,5].

The clinical presentation of BPF is predominantly pulmonary, with abdominal symptoms being less frequent. Bilioptysis, the coughing up of bile, is a key clinical feature, affecting 12.5 to 77.8 % of patients as per various studies. Other signs are dominated by vomica, which indicates the rupture of the hepatic hydatid cyst into the lung. Hepatobiliary signs such as jaundice are not specific [5]. It's crucial to highlight that these symptoms can significantly vary among patients, necessitating a thorough clinical evaluation for precise diagnosis and treatment planning. Imaging is invariably crucial, and each imaging modality offers its unique advantages.

Findings from chest X Rays can be typical or atypical; frequently, an opacity in the lower lobe or a pleural effusion is observed. An abdominal ultrasound can be beneficial by delineating the liver's morphology and potential obstructions in the biliary tree.

Computed Tomography (CT) scans assist in differentiating the relationships among the cyst, blood vessels, and bile ducts. It also aids in assessing local extension and identifying a BBF and other potential sites of the disease.

The information provided by these imaging studies plays a pivotal role in determining the most appropriate therapeutic planning.

The goal of treating broncho biliary fistulas is to close the fistula and address its root cause, typically requiring surgical intervention along with appropriate preoperative preparation.

This preparation involves controlling the infection with tailored antibiotic therapy, ensuring effective respiratory physiotherapy, and rebalancing hydro electrolytes and caloric intake.

The surgical approach primarily targets five key objectives: first, it addresses the treatment of endothoracic lesions. Second, it involves the management of hepatic lesions following hepato-diaphragmatic disconnection, accomplished through either total or partial pericystectomy. This method is favored over hepatic resection as it prevents unnecessary loss of healthy parenchyma while preserving tissue conducive to healing and regeneration. Third, the approach includes the detection and treatment of biliary fistulas. Fourth, it focuses on repairing the diaphragm. Lastly, the approach ensures adequate drainage of the pleural and hepatic cavities when necessary [5].Surgical access involved either a laparotomy, thoracotomy, or a thoracoabdominal (TA) incision **.**The selection of the access route and surgical technique should not rely on the surgeon's preference but should instead be customized to suit the specific requirements determined by the location, nature, and extent of the disease, particularly focusing on the type of broncho biliary fistula (FBB), along with thorough preoperative radio clinical and biological assessments [6]**.**

A Thoracoabdominal (TA) incision provides optimal exposure to both thoracic and hepatic lesions simultaneously, with an acceptable rate of morbidity and mortality. This approach is particularly valuable in ensuring wide surgical access. It offers enhanced comfort and safety when addressing major pulmonary parenchymal lesions that often require controlled resections, such as lobectomy or segmentectomy. However, left-sided biliobronchial fistulas present unique challenges. Hepatodiaphragmatic disconnection via an exclusive left thoracic approach is technically demanding due to the complex anatomical relationships between the left hepatic lobe and adjacent organs, notably the stomach, increasing the risk of iatrogenic injury [5].In cases of active biliobronchial fistulas with high-output drainage, achieving fistula closure necessitates hepatobronchial disconnection. This step requires prior verification of unobstructed bile duct flow, making laparotomy indispensable to confirm and secure biliary drainage [6].Laparoscopy is increasingly used in the surgical management of hydatid cysts, given its advantages in reducing postoperative morbidity. However, it remains an unvalidated approach for complicated cases, particularly those involving double biliary and bronchial fistulas. In such situations, laparoscopy carries a higher risk of hydatid fluid spillage, which may increase the likelihood of recurrence [7,8].Postoperative complications are varied, with septic events being the most common owing to the intrinsically contaminated nature of biliobronchial fistula (BBF) surgery. Reported postoperative morbidity and mortality rates remain significant, ranging from 12.2 % to 50 % [5].

In our patient, the hydatid cyst of the left hepatic lobe, complicated by a biliobronchial fistula, was successfully managed via laparotomy alone. Based on Mesteri's classification [4], types IA and IB can be treated without thoracotomy. Our patient had type IB BBF and this approach allowed for simultaneous treatment of thoracic, diaphragmatic, and abdominal lesions in a single operative session, thereby reducing hospital stay and overall costs, with favorable immediate and long-term postoperative outcomes.

This case suggests that with careful imaging and interdisciplinary assessment, an abdominal approach alone may be a safe and sufficient option for selected left-sided BBF patients, even in the absence of thoracic intervention.

## 4 Conclusion

Broncho biliary fistula is a rare and hazardous complication of hepatic hydatid cysts, predominantly affecting the right lobe of the liver. Left-sided localization is uncommon and poses unique challenges, necessitating precise evaluation and a well-established therapeutic strategy. This case highlights the importance for both surgeons and pulmonologists to consider the possibility of an underlying ruptured pulmonary hydatid cyst in patients presenting with left-sided pneumonia. Although rare, this diagnosis should not be overlooked, as early identification and appropriate management can significantly improve patient prognosis.

## Consent

Informed consent for publication of information and images was provided by the patient.

## Ethical approval

It is exempt from ethical approval because it is an observation report.

## Funding

No funding.

## Author contribution

AS, MBK = Study concept, Data collection, and surgical therapy for the patient.

AS, FJ, MG = Writing- original draft preparation.

AS, MBK, WEG = Editing and writing.

MB = Senior author and manuscript reviewer.

## Guarantor

Sarraj Achref.

## Research registration number

Not applicable.

## Conflict of interest statement

The authors declare that no funds, grants, or other support were received during the preparation of this manuscript.

The authors have no relevant financial or non-financial interests to disclose.
